# Heart failure monitoring with a cardiac resynchronization therapy device-based cardiac contractility sensor: a case series

**DOI:** 10.1186/1752-1947-8-27

**Published:** 2014-01-27

**Authors:** Jacques Mansourati, Mélanie Heurteau, Jérôme Abaléa

**Affiliations:** 1 Department of Cardiology, University Hospital of Brest, Hôpital de la Cavale Blanche, Boulevard Tanguy Prigent, 29609 Brest, France; 2 EA 4324, ORPHY, Université de Bretagne Occidentale, 3 Rue des Archives, 29238 Brest, France; 3 Sorin CRM SAS, Avenue Réaumur, 92140 Clamart, France

**Keywords:** Heart failure, Monitoring, Cardiac resynchronization therapy, Peak endocardial acceleration, SonR, Sensor

## Abstract

**Introduction:**

The SonR signal has been shown to reflect cardiac contractility. It is recorded with an atrial lead connected to a cardiac resynchronization therapy defibrillator. For the first time, clinical evidence on the use of the SonR signal in the monitoring of the clinical status of heart failure patients implanted with cardiac resynchronization therapy defibrillator are presented through three clinical cases.

**Case presentation:**

In the two first patients (non-Hispanic/Latino white), the SonR amplitude increases concomitantly to clinical status improvement subsequent to cardiac resynchronization therapy defibrillator implantation. In the third patient (non-Hispanic/Latino white), a decrease in SonR amplitude is observed concomitantly to atrial fibrillation and clinical status deterioration.

**Conclusions:**

This case series reports the association between SonR signal amplitude changes and patients’ clinical status. Combined with remote monitoring, early SonR signal amplitude remote monitoring could be a promising tool for heart failure patients’ management.

## Introduction

The peak endocardial acceleration signal, or SonR signal has been shown to correlate with the first heart sound [1], supporting the hypothesis that the SonR signal amplitude correlates with left ventricular (LV) dP/dt_max_, a measure of the contractile function of the heart [2-5] and may be a useful tool to monitor cardiac function. In order to further assess this hypothesis, we evaluated the association between SonR signal amplitude and patients’ clinical status through the three following case reports.

The hemodynamic SonR sensor (SonR™ sensor, Sorin CRM, Clamart, France) is a micro-accelerometer encapsulated in a hermetic can inside the tip of an atrial pacing lead (SonR™ sensor, Sorin CRM, Clamart, France). The SonR signal is monitored and computerized by the embedded SonR algorithm through an implantable system consisting of an atrial pacing lead (SonRTip™ lead, Sorin CRM, Clamart, France) connected to a cardiac resynchronization therapy defibrillator (CRT-D) device. Additionally, SonR allows automatic weekly atrioventricular (AV) and interventricular (VV) delays optimization in heart failure (HF) CRT-D patients, at rest and exercise [6-9].

The three following clinical cases report information on recipients of Paradym RF™ SonR CRT-D (Sorin CRM SAS, Clamart, France), implanted for HF with either ischemic (ICM) or dilated cardiomyopathy (DCM).

## Case presentation

**Figure 1:**
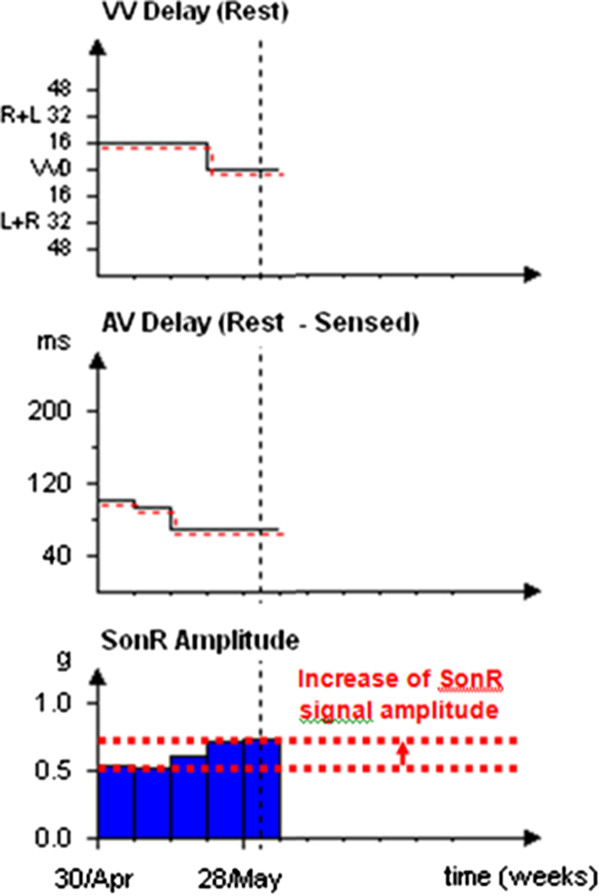
Case no. 1 - SonR signal amplitude with early improvement after cardiac resynchronization therapy defibrillator device implantation.

The first patient is a 70-year-old man (non-Hispanic/Latino white) with an ICM. Two myocardial infarctions occurred in 1986 and 1997, followed by a coronary artery bypass grafting (CABG) in 1997. He was resuscitated from a cardiac arrest related to graft occlusion in 2003. A first single-chamber implantable cardioverter-defibrillator (ICD) was implanted in 2006 (MADIT II indication). In 2012, the patient became severely symptomatic (New-York Heart Association (NYHA) class III) with a left bundle branch block (LBBB) and a left ventricular ejection fraction (LVEF) of 26%. Therefore, the single chamber ICD was upgraded to the CRT-D device. Within three weeks, the AV and VV delays were automatically adjusted by the device on a weekly basis; the patient became asymptomatic while the SonR signal amplitude increased (Figure 1). No concomitant therapy changes occurred during this period.

**Figure 2:**
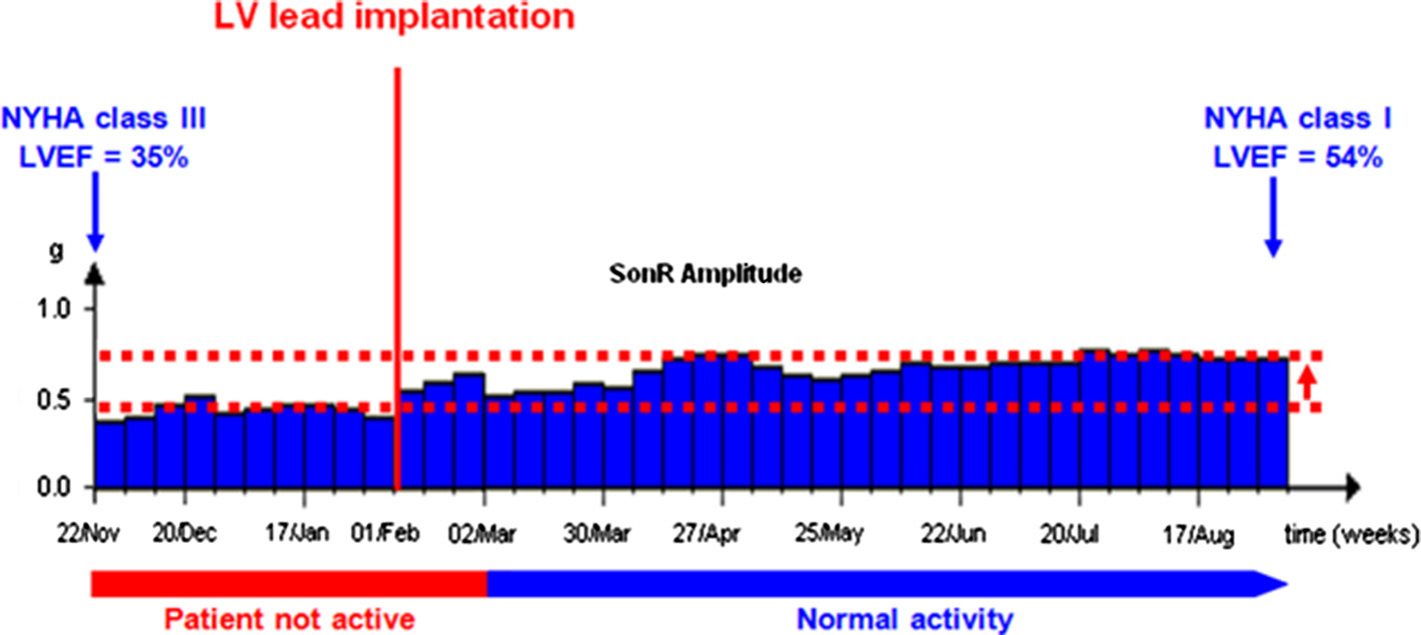
Case no. 2 - SonR signal amplitude evolution before and after cardiac resynchronization therapy defibrillator pacing.

The second patient is a 65-year-old woman (non-Hispanic/Latino white) who presented with a DCM related to radiotherapy and chemotherapy (left-side breast cancer treated in 2007). She was in NYHA class II/III, with a LVEF of 35% and a LBBB. The patient was implanted with the CRT-D device from the right thoracic side in November 2011. However, the first attempt of LV lead implantation was unsuccessful and ventricular pacing was prevented until the second attempt, which succeeded in February 2012. Subsequently, the patient progressively improved (NYHA class I on September 2012). Figure 2 shows the SonR signal amplitude evolution during these two periods (before and after LV lead implantation): the amplitude increased while the patient’s status improved after the successful LV lead implantation.

**Figure 3:**
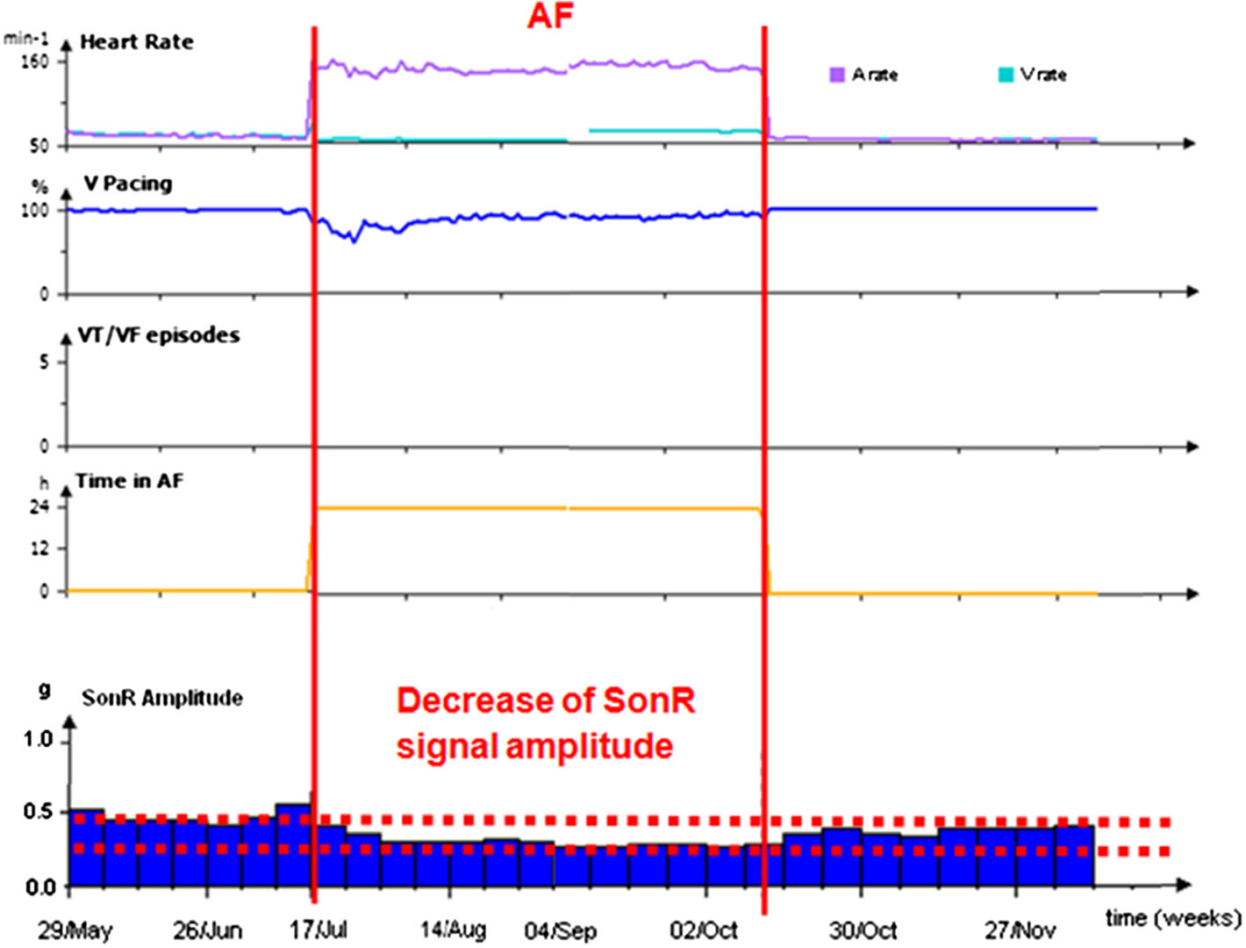
Case no. 3 - SonR signal amplitude evolution in the presence of atrial fibrillation and partial loss of biventricular capture.

A third 74-year-old man (non-Hispanic/Latino white) underwent CABG in 1993. In 2005, he experienced atrial fibrillation (AF) which resulted in HF. After treatment with amiodarone, sinus rhythm (SR) was restored and the patient became asymptomatic with a LVEF of 12% and LBBB. In 2005, the patient was implanted with a prophylactic single chamber ICD. In 2012, the patient became severely symptomatic (NYHA class III) with LBBB and a LVEF of 20%. Based on this evolution, the patient was upgraded with the CRT-D device, in May. During an outpatient visit on July 17, an electrocardiogram (ECG) revealed AF. The device diagnosis showed that AF started on July 16 with a mode switch to VVI 80bpm. Both ventricles were only captured 90% of the time. On July 26, the patient was hospitalized for HF and his HF treatment was modified. On September 9, the patient was in NYHA class I and his SR was restored by electrical cardioversion on October 10. No therapy changes occurred during this period. Figure 3 shows the decrease of the SonR signal amplitude that started concomitantly to AF and remained low as long as AF lasted. Following sinus rhythm restoration on October 10, the SonR signal amplitude started to increase, reaching its initial value after the persistence of the sinus rhythm.

Therefore, an association between the SonR signal amplitude change and patients’ clinical status based on NYHA was observed in these three clinical cases.

## Discussion

This report is the first to describe SonR signal amplitude changes according to patients’ clinical status. In the two first patients, the SonR amplitude increases concomitantly to clinical status improvement after CRT-D implantation. In the third patient, a decrease in SonR amplitude is observed concomitantly to an atrial fibrillation episode and clinical status deterioration.

Further adjustments to the SonR device could be of benefit to the monitoring process of CRT-D patients: (1) while AV and VV delays optimization are automatically performed weekly by SonR, displaying daily SonR amplitude values would be preferable for monitoring purposes; (2) finally, the percentage of modification in clinical status could be quantified for the development of an alert.

Combined with remote monitoring, which is now widely accepted based on accumulating evidence that outcomes are better compared with standard in-clinic follow-up, early SonR signal amplitude remote monitoring could be a promising tool for HF patients’ management. Several recent trials on HF monitoring based on device diagnostic information confirmed the difficulty to monitor HF using a sole indicator and suggested the need to incorporate multiple parameters [10]. In this context, the SonR signal amplitude could be combined with other device-based parameters in order to improve the accuracy of the diagnostic. However, technical improvements and outcome prospective trials are warranted.

## Consent

Written informed consent was obtained from the patients for publication of this case series and any accompanying images. A copy of the written consents is available for review by the Editor-in-Chief of this journal.

## Abbreviations

AF: atrial fibrillation; AV: atrioventricular; CABG: coronary artery bypass grafting; CRT-D: cardiac resynchronization therapy defibrillator; DCM: dilated cardiomyopathy; HF: heart failure; ICD: implantable cardioverter-defibrillator; ICM: ischemic cardiomyopathy; LBBB: left bundle branch block; LVEF: left ventricular ejection fraction; NYHA: New York Heart Association; SR: sinus rhythm; VV: interventricular.

## Competing interests

Jacques Mansourati declares consultant fees and research fees with Biotronik, Boston Scientific, Medtronic, St Jude Medical and SORIN Group. Melanie Heurteau is employed by Sorin Group. Jerome Abalea declares no competing interests.

## Authors’ contributions

JM and JA analyzed and interpreted the patient data regarding the heart failure disease and the signal analysis. All authors read and approved the final manuscript.
